# Accuracy of a Chatbot (Ada) in the Diagnosis of Mental Disorders: Comparative Case Study With Lay and Expert Users

**DOI:** 10.2196/13863

**Published:** 2019-10-29

**Authors:** Stefanie Maria Jungmann, Timo Klan, Sebastian Kuhn, Florian Jungmann

**Affiliations:** 1 Department of Psychology, Johannes Gutenberg-University Mainz Mainz Germany; 2 University Medical Center, Johannes Gutenberg-University Mainz Mainz Germany

**Keywords:** artificial intelligence, eHealth, mental disorders, mHealth, screening, (mobile) app, diagnostic

## Abstract

**Background:**

Health apps for the screening and diagnosis of mental disorders have emerged in recent years on various levels (eg, patients, practitioners, and public health system). However, the diagnostic quality of these apps has not been (sufficiently) tested so far.

**Objective:**

The objective of this pilot study was to investigate the diagnostic quality of a health app for a broad spectrum of mental disorders and its dependency on expert knowledge.

**Methods:**

Two psychotherapists, two psychology students, and two laypersons each read 20 case vignettes with a broad spectrum of mental disorders. They used a health app (Ada—Your Health Guide) to get a diagnosis by entering the symptoms. Interrater reliabilities were computed between the diagnoses of the case vignettes and the results of the app for each user group.

**Results:**

Overall, there was a moderate diagnostic agreement (kappa=0.64) between the results of the app and the case vignettes for mental disorders in adulthood and a low diagnostic agreement (kappa=0.40) for mental disorders in childhood and adolescence. When psychotherapists applied the app, there was a good diagnostic agreement (kappa=0.78) regarding mental disorders in adulthood. The diagnostic agreement was moderate (kappa=0.55/0.60) for students and laypersons. For mental disorders in childhood and adolescence, a moderate diagnostic quality was found when psychotherapists (kappa=0.53) and students (kappa=0.41) used the app, whereas the quality was low for laypersons (kappa=0.29). On average, the app required 34 questions to be answered and 7 min to complete.

**Conclusions:**

The health app investigated here can represent an efficient diagnostic screening or help function for mental disorders in adulthood and has the potential to support especially diagnosticians in their work in various ways. The results of this pilot study provide a first indication that the diagnostic accuracy is user dependent and improvements in the app are needed especially for mental disorders in childhood and adolescence.

## Introduction

### Background

Digital media have become enormously important in the health sector. Up to 80% of the internet users inform themselves on the Web about health [1], and about 60% of patients search for their symptoms on the internet before or after a visit to the doctor [2]. Experts estimate that there are over 380,000 health-related mobile apps worldwide [3].

Health apps play an important role not only in physical diseases but also particularly in mental health conditions and disorders [4-6]. For mental disorders, access to professional diagnosis and treatment is often difficult and delayed (eg, long waits and concerns about psychotherapy). In addition, there is considerable uncertainty in the population about the significance of the symptoms (eg, at what point feelings and behaviors are pathological). The advantage of health apps is low-threshold, locally and temporally flexible, and cost-efficient access [4]. The services are independent of the medical care situation, can be individually adapted and integrated into everyday life, and increase the self-help potential [5,7]. A systematic literature review showed that especially people who felt stigmatized by their problem or ashamed of it (eg, encopresis and eating disorders) use electronic mental health (e–mental health) [8]. Digital media can also be highly relevant for certain target groups. In mental disorders in childhood, for example, there are more possibilities for nonverbal recording of symptoms, and parents can be supported in coping with problems in everyday life [9]. As young people use new media every day (97% daily internet consumption), and mental health problems at this age are usually experienced as stigmatizing and shameful, the youth are considered particularly accessible to health apps [10]. In addition, health apps are promising for patients with chronic or recurrent phases of illness, which are particularly common in mental disorders.

In recent years, an enormous number of health apps have been developed for mental health conditions and disorders, the number of which is now hardly manageable. The proportion of health apps for mental health is about 29% of all health apps worldwide [11]. Health apps for mental health cover various areas of health promotion, prevention, screening and diagnostics, management, treatment, and aftercare [12]. These apps are usually aimed at consumers, that is, people suffering from symptoms. Recent developments also target professionals and, more recently, the public health care system (eg, pilot function and screening) [13,14].

Given the large number of health apps, the problem arises that they are used extensively but are usually not (sufficiently) evaluated and tested. Several reviews [15-18] have found that health apps for mental health have rarely been tested for their usefulness and effectiveness and often have ethical and legal shortcomings (eg, data privacy and safety). For example, Wisniewski et al [15] found that 15% to 45% of studied apps for anxiety, depression, and schizophrenia made medical claims, although these were rarely evidence-based, and no apps had Food and Drug Administration marketing approval. In addition, only 50% to 85% included a privacy policy [15]. Even if the apps have many benefits as described above, health-related internet use can also have negative or harmful effects on one’s emotional state and health behavior, as research shows, for example, on the phenomenon of cyberchondria [19]. Cyberchondria refers to an excessive health-related internet search resulting in an increase in emotional distress and health anxiety (eg, because of ambiguous information or serious disease [19]).

There is a particularly great need for research into apps for the screening or diagnosis of mental disorders [5]. This gap in research contrasts with the importance that diagnostic or screening tools can have, for example, in assigning patients to appropriate medical disciplines and practitioners.

### Health Apps for Screening and Diagnosis of Mental Disorders

Regarding diagnostics using e–mental health, a distinction is made between the collection of objective and subjective data [5,20]. Objective data (mostly psychophysiological measures or behavioral activity) are recorded via sensors in or connected to the mobile phone or so-called wearables. For example, Valenza et al [21] showed that heart rate variability predicted mood swings in patients with a bipolar spectrum disorder. So far, there are few empirical findings on the use of wearables in mental disorders; only about 1.5% of studies on wearables deal with mental health [22]. A recent systematic review showed that objective data were promising in predicting moods and mood changes, but much more empirical evidence was needed to reliably evaluate potentials and risks [20].

There are countless health apps that assess subjective data, such as apps used for assessments (eg, Web-based questionnaires) or tracking (eg, monitoring mood or medication via diaries) [23]. Regarding self-report instruments that were adapted into a mobile phone app, there are few evaluated Web-based questionnaires on depression and posttraumatic stress disorder that showed a psychometric quality comparable with the paper-pencil version [23-25]. Some apps, such as Moodpath [26], include questions based on the operationalized diagnostic criteria of the International Classification of Diseases (ICD), tenth revision [27]. In Moodpath, users are asked different questions 3 times a day for 14 days according to the diagnostic criteria for depressive disorders. On the basis of the indicated symptom patterns, an algorithm determines possible depression (screening) and makes an assessment of severity. The results of diagnostic apps are often based on algorithms or artificial intelligence (AI), which means that computers can simulate complex human cognitions and actions.

Regarding mental tracking, a few apps on mood and affective disorders have been empirically investigated. For example, Hung et al [28] found in patients with depression that daily data on depression, anxiety, and sleep quality in a mobile phone app were significantly related to clinician-administered depression assessment at baseline. For bipolar affective disorder, a mobile phone app identified lower physical (location changes recorded via global positioning system) and social (outgoing messages) activities as significant predictors for increased depressive symptoms and lower physical but increased social activity for increased manic symptoms [29].

In contrast to apps for physical diseases (eg, Ada—Your Health Guide [30] and IBM Watson Health [31]), apps for mental health focus almost exclusively on a single symptom or single mental disorder, rather than on a broader spectrum. However, especially for the purpose of screening, it seems interesting and necessary at all 3 levels (eg, individual, practitioner, and public health system) that a single app asks for a variety of symptoms and mental disorders and provides information about the range of psychopathology. Only a few apps for mental health, such as WhatsMyM3 [32] (anxiety, depression, bipolar affective disorder, and posttraumatic stress) and T2 Mood Tracker [33] (anxiety, depression, head injury, and posttraumatic stress), assess multiple mental health conditions. However, these are usually limited to anxiety-depressive symptoms and have so far been little evaluated [23]. Therefore, in this study we used a medicine app that covers a wide range of physical and mental health conditions.

The aim of this pilot study was to test for the first time the diagnostic agreement of a medicine app and case vignettes over a broad spectrum of mental disorders. We expected at least moderate diagnostic agreement (ie, interrater reliability Cohen kappa≥0.41; hypothesis 1). As health apps are used both as a self-assessment at the consumer level and a diagnostic support system by experts and practitioners [34,35], we examined the diagnostic quality, depending on the user’s level of expert knowledge (ie, 3 user groups: psychotherapists, psychology students, and laypersons). Given the less advanced state of development of diagnostic health apps for mental health than for physical diseases [5,36], we hypothesized that diagnostic accuracy for mental disorders is dependent on expert knowledge (eg, symptom checker includes fewer psychiatric terms, and alternative terms need to be entered; hypothesis 2).

## Methods

### Design

**Figure 1:**
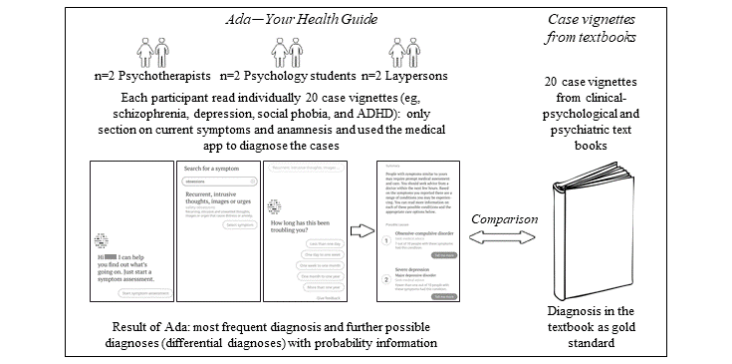
Method and procedure of the study. ADHD: attention-deficit hyperactivity disorder.

A health app (Ada—Your Health Guide [30]) was used to diagnose 20 case vignettes from well-known textbooks of psychiatry and clinical psychology [37-40] by 3 groups: psychological psychotherapists, psychology students, and persons from the general population without previous professional knowledge of mental disorders (laypersons). Figure 1 illustrates the design and method.

### Participants

Table 1 shows the sociodemographic characteristics of the participants.

**Table 1:** Sociodemographic characteristics of the participants, subdivided into psychotherapists, psychology students, and laypersons.

Characteristics	Psychotherapists (n=2)	Psychology students (n=2)	Laypersons (n=2)	Statistics	
				*P* value	Effect size
Age (years), mean SD	40.0 (11.3)	22.0 (4.2)	40.0 (8.5)	.19	η^2^_p_=0.67
Sex, female, n (%)	1 (50)	1 (50)	1 (50)	>.99	Φ=0
Occupation, mean (SD) or with or without vocational training	14.0 (7.1) years professional experience^a^	4.0 (4.2) number of semesters	n=1 data scientist; n=1 without vocational training	—^b^	—^b^

^a^One of both is additionally a child and adolescent psychotherapist (first author).

^b^No comparative statistics possible due to different occupations.

### Instruments

#### Health App for Diagnosis

Ada—Your Health Guide [30] is a Conformité Européenne–certified (ensures safe products within the European economic area) health app for the screening and diagnostic support of health conditions, primarily for physical diseases but increasingly also for mental health conditions and disorders. This app can be used both at the consumer level as a self-assessment app and by experts and practitioners as a diagnostic decision support system [35]. On the basis of AI, the chatbot asks for existing complaints adaptively and analogously to a medical or psychotherapeutic anamnesis interview. The Ada chatbot [30] is based on a medical database with constantly updated research findings. As a result, the diagnosis is determined that best matches the pattern of symptoms entered. The user is given a probability of a possible diagnosis and, as differential diagnoses, other less probable diagnoses (eg, 8/10 people with the symptoms described suffer from a depressive disorder). Patients or relatives (eg, parents and caregivers) receive an assessment of the urgency of seeking medical advice. Ada—Your Health Guide [35] was selected to investigate the above research questions for the following reasons (also in comparison with other symptom checkers [41,42]): (1) in addition to somatic symptoms, the app considers a wide spectrum of mental health conditions; (2) the app provides probabilities of possible and differential diagnoses (indications of comorbidities); (3) the app is widespread (>5 million users in >130 countries), publicly available, and free; (4) available in different languages (including English and German), and (5) in comparison with other symptom checkers (eg, Your.MD [43] and Babylon Health [44]), Ada provided more accurate diagnoses [42,45].

#### Knowledge of Mental Disorders

The user’s knowledge of mental disorders was assessed on a 5-point Likert scale (1=not at all to 5=very good).

#### Procedure

After the informed consent, participants were instructed to carefully read the case vignette and then use the app to determine a diagnosis. A total of 20 case vignettes from psychiatry and clinical psychology textbooks were used, with 12 cases from adulthood [37,39] and 8 cases from childhood and adolescence [38,40]. All participants worked on one case after the other. The case vignettes were selected in such a way that a broad spectrum of mental disorders could be examined (see a list of mental disorders in Multimedia Appendix 1). The case vignettes included the initial symptoms before treatment (reason to seek treatment) and the anamnestic information, without naming or citing the diagnosis. The participants worked on the case with the health app on a tablet. The study duration was 3 to 6 hours per participant, divided into 2 to 3 individual sessions (most of the time was spent reading the 20 case vignettes). The participants (except the psychotherapists) received financial compensation (€10/hour) or a course credit (students).

### Data Analysis

The main outcome was the agreement between the main diagnosis of the case vignette in the textbook and the result given by the app (the most probable diagnosis). Consistent labeling of the mental disorders was considered when assessing agreement. As an exception, the terms abuse and addiction were judged to agree, as the app did not distinguish between abuse and addiction to our knowledge. The diagnoses were compared at the level of 4-digit codes in the ICD (eg, anxiety disorders such as social anxiety and agoraphobia or personality disorders such as borderline personality disorder). If the 4th digit represents a more detailed specification (eg, obsessive-compulsive disorder: predominantly obsessive-compulsive behavior and thoughts or severity of the depressive episode), the 3-digit code match was counted (for the following disorders: depressive disorder, bipolar affective disorder, obsessive-compulsive disorder, conduct disorder, or schizophrenia). To consider the function and purpose of the screening and diagnostic app (eg, further diagnostic procedures required), no distinction was made between the subtypes of dementia (eg, Alzheimer and vascular dementia) and that of urinary incontinence (eg, stress incontinence and enuresis diurnal or nocturnal). The list of diagnoses in the textbooks and the results from the app can be found in Multimedia Appendix 1. The statistical outcomes were calculated as the percentage of agreement and the Cohen kappa coefficient (interrater reliability) for controlling random agreements. According to Landis and Koch [46], kappa values between 0.41 and 0.60 can be rated as moderate, between 0.61 and 0.80 as good, and >0.81 as very good. The agreement was checked if the secondary or differential diagnosis given by the app was also included (eg, bipolar disorder in the textbook but as a differential diagnosis in the app). Cohen *d* was calculated as effect size for group differences and partial eta-square for variance analyses. All statistical analyses were conducted using SPSS version 23 (IBM SPSS) [47], with an alpha level of .05. Following the study by Field [48], the Ryan, Einot, Gabriel, and Welsch Q procedure was used in post hoc tests to control the alpha error (same sample size; the Gabriel procedure was used when the sample sizes were different).

## Results

### Knowledge of Mental Disorders

Self-rated knowledge of mental disorders varied significantly depending on the group (ie, psychotherapists, students, or laypersons)—*F*_2,3_=18.50; *P*=.02; partial eta-square=0.93. Post hoc analyses indicated that laypersons (mean 1.50, SD 0.71) reported significantly lower knowledge than students (mean 3.50, SD 0.71; *P*=.04) and psychotherapists (mean 5.00, SD 0; *P*=.01), with the last 2 groups having a marginally significant difference from each other (*P*=.08).

### Percentage Agreement and Interrater Reliability

For mental disorders in adulthood, we found for the 72 case records (6 users×12 mental disorders), a percentage agreement of 68% and an interrater reliability according to Cohen kappa 0.64 between the textbook diagnosis and the result produced by the app. Taking into account the differential diagnoses, we found a percentage agreement of 85% and Cohen kappa 0.82. For mental disorders in childhood and adolescence, 48 case records (6 users×8 mental disorders) showed a percentage agreement of 42% (including differential diagnoses: 56%) and a Cohen kappa 0.40 (including differential diagnoses: kappa=0.52).

Table 2 shows the mean number (n), percentage (%), and Cohen kappa coefficients, differentiated among the 3 different user groups (ie, psychotherapists, students, and laypersons).

For mental disorders in adulthood, the Cohen kappa values were 0.78 (95% CI 0.60-0.95) for psychotherapists, 0.55 (95% CI 0.35-0.76) for students, and 0.60 (95% CI 0.39-0.80) for laypersons. Regarding case vignettes from childhood and adolescence, Cohen kappa values were numerically higher for psychotherapists (kappa=0.53, 95% CI 0.28-0.77) than for students (kappa=0.41, 95% CI 0.18-0.63) and laypersons (kappa=0.29, 95% CI 0.08-0.49).

Multimedia Appendix 1 lists the 20 mental disorders of the case vignettes as well as the main diagnoses in Ada Health and examples of differential diagnoses. The app mostly identified the main diagnosis (67% [8/12] of cases for adulthood and 44% [3.5/8] of cases for childhood and adolescence); it reported the differential diagnoses in an additional 17% (2/12) of cases for adulthood and 13% (1/8) of cases for childhood and adolescence. If the differential diagnoses are included, all diagnoses except undifferentiated somatization disorder, separation anxiety, and selective mutism in childhood were correctly detected.

**Table 2:** Mean number, percentage, and Cohen kappa coefficients for agreement between the textbook diagnosis and the result from Ada Health.

Case reports	Main diagnosis in Ada Health						Additional consideration of differential diagnoses in Ada Health					
	Psychotherapists		Students		Laypersons		Psychotherapists		Students		Laypersons	
	n (%)	kappa	n (%)	kappa	n (%)	kappa	n (%)	kappa	n (%)	kappa	n (%)	kappa
Adulthood (n_max_=12)	9.5 (79)	0.78	7 (58)	0.55	7.5 (63)	0.60	11 (92)	0.91	10.5 (88)	0.87	8.5 (71)	0.69
Childhood and adolescence (n_max_=8)	4.5 (56)	0.53	3.5 (44)	0.41	2.5 (31)	0.29	4.5 (56)	0.59	5 (63)	0.52	4 (40)	0.45

### Number of Questions and Duration

To find a solution, the app had to ask an average of 34 questions per case (mean 33.78, SD 8.73) about the type and duration of the symptoms. There was no significant difference between the groups (*F*_2,117_=1.89; *P*=.16; partial eta-square=0.03). The average time to complete was 409 seconds (SD 141.23). The groups differed in the average time for completion (*F*_2,96_=9.93; *P*<.001; partial eta-square=0.17). Psychotherapists (mean 457.28, SD 138.61) and students (mean 415.82, SD 143.11), who did not differ from each other (*P*=.40), showed a significantly longer time for completion than the laypersons (the time recorded for only 1 layperson; mean 299.45, SD 141.23; *P*<.001).

## Discussion

### Principal Findings

In this pilot study, we tested whether a health app (Ada—Your Health Guide [30]) could detect mental disorders in children, adolescents, and adults. A total of 3 groups of users (ie, psychotherapists, psychology students, and laypersons) used the app to diagnose 20 case vignettes. Across all users, the agreement between the textbook diagnoses and the app was moderate (kappa=0.64) for mental disorders in adulthood and low (kappa=0.40) for that in childhood and adolescence. Adding differential diagnoses, good (kappa=0.82) and moderate (kappa=0.52) values, respectively, were obtained for interrater reliability.

When psychotherapists applied the app, there was a good agreement (kappa=0.78) between the results of the app and the diagnoses in the textbook on mental disorders in adulthood. This value is comparable with interrater reliabilities between 2 psychologists for diagnoses assessed with structured clinical interviews (kappa=0.71 for Axis I disorders and kappa=0.84 for personality disorders [49]). The diagnostic agreement was moderate (kappa=0.55/0.60) when students and laypersons used the app. The addition of differential diagnoses showed a good to very good interrater reliability (kappa=0.69-0.91). In 17% of the cases, the app did not give the diagnosis as the main diagnosis but as a differential diagnosis. Although the app assessed a different diagnosis as more likely, the main diagnosis of the case report was considered in some cases as a differential diagnosis.

For mental disorders in childhood and adolescence, a moderate diagnostic quality was found when psychotherapists (kappa=0.53) and students (kappa=0.41) used the app, whereas the quality was low for laypersons (kappa=0.29). In contrast to mental disorders in adulthood, the addition of differential diagnoses improved the diagnostic quality in childhood and adolescence to a lesser extent.

Taken together, only for mental disorders in adulthood, and when psychotherapists used the app, did Ada—Your Health Guide show good diagnostic quality. The app can serve as an indication of a mental health problem in the range of moderate agreement (adult mental disorders: students and laypersons; child and adolescent mental disorders: psychotherapists, students). With an average app time of 7 min, the app can be an efficient tool for the initial evaluation and screening of mental health problems and disorders. So, this pilot study indicates that expert knowledge tends to lead to better diagnostic quality when using the health app.

When comparing mental disorders in adulthood and childhood and adolescence, the app shows deficits for mental disorders in children and adolescents. For example, the app could not detect separation anxiety in childhood or selective mutism in any operation. On the one hand, this may be because of deficits in the app, on the other, mental disorders in childhood and adolescence are more often characterized by less specific symptom descriptions—children and adolescents show fewer specific symptoms and, from a developmental perspective, more frequent temporary subclinical symptoms [50]. This may also have led to confusion with the concrete naming and focusing of symptoms in childhood and adolescence. Examples include case reports on attention-deficit hyperactivity disorder (ADHD) and separation anxiety. In the ADHD case vignette, fears are mentioned first (eg, *would see ghosts*) [40]. In the case of separation anxiety, the initial focus is on describing the problematic relationship of the parents. In both cases, the hallmarks of the disorders are reported later and relatively profoundly. In addition, the app [30] may not include relevant terms and psychopathological characteristics, such as school fear and selective mutism. There is a clear need to catch up here. Especially in the case of enuresis, the results generated by the app, such as *mixed incontinence* or *stress incontinence*, made it clear that these were primarily terms pertaining to adults. As Ada—Your Health Guide [30] is based on a medical database with updated research findings, these deficits in the detection of mental disorders can also be because research activity in children and adolescents is significantly lower than that in adults. In the case of disorders with somatic symptoms (eg, undifferentiated somatization disorder), the diagnosis was more difficult because of the delimitation of psychological and physical symptoms. The overall interrater reliability in this study is lower than in studies that use structured clinical interviews [49].

It is important to consider the aims of screening and diagnostic apps. Health apps (eg, Ada—Your Health Guide [30]) do not aim to replace doctors or psychotherapists. Psychopathological symptoms can only be adequately understood and classified by a detailed anamnesis, the consideration of the temporal course, and the correct assessment of inclusion and exclusion criteria. For example, a severe, recurrent depressive disorder or multiple comorbidities worsen prognosis and require treatment (eg, combined treatment with psychotropic drugs) different from more circumscribed cases, such as a mild and single depressive episode. To our knowledge, there is currently no diagnostic app that captures this complexity (especially several comorbidities). Furthermore, the benefits of personal interaction should not be underestimated, as some behavioral abnormalities become apparent especially in direct contact (eg, hyperactivity or personality disorders), and unintended or intentional bias tendencies (eg, social desirability) can be more easily identified. Therefore, we consider the clarification of problems and diagnostics by experts to be of immense importance. The evaluated diagnostic apps should rather be regarded as low-cost, low-threshold, and time-efficient support in the diagnosis of mental disorders in adulthood [5]. There is great potential for the application of AI-supported diagnostics at the level of the consumer or patient, the experts, and the health care system, for example the following [14]:

- *Consumers and patients:* for example, screening of symptoms, combined with possible emotional relief for the affected person (eg, diagnosis as an explanation or treatment option) and a recommendation for action (eg, seeking medical advice).
- *Professionals:* for example, support in more efficient exploration and diagnosis (eg, bringing the result of the health app to the initial consultation), consideration and explanation of differential diagnoses, rapid reaction to significant symptoms (eg, suicidal intentions and alcohol consumption), and support in making indication decisions.
- *Macro/health care system:* for example, optimizing the assignment to treatment providers or treatment settings, supporting employees of other occupational groups in the health care system.

### Limitations and Research Perspectives

In this study, the health app was only tested on case vignettes, and the user groups had a very small sample size. This limits the transferability of our results to everyday practice (low ecological validity). In addition, in the case of small samples, the performance of individual and outlier values plays a major role [51]. A recent study examined another symptom checker (Babylon Health [44]) that had comparable methodological limitations (case vignettes and small sample [52]). In contrast to this study, we investigated mental disorders for which the apps have so far been little developed, requiring a first pilot study. In addition, we focused on the question of whether the diagnostic quality is dependent on expert knowledge and examined the quality when experts, students, and laypersons used the app.

A next step will be to investigate the diagnostic accuracy of health apps for mental disorders in a direct interaction of practitioner and patient and with a larger sample. Depending on the research question, the design has to be differentiated. If the diagnostic quality is of interest, the agreement of the results of the app applied by the end user or patient could be compared with the current gold standard for the diagnosis of mental disorders, that is, structured or standardized interviews (eg, Diagnostic Interview for Mental Disorders [43]). If investigating the question of how well the health app can support clinicians in diagnosing mental disorders, the comparison of the clinical diagnosis with and without an additional health app should be examined. It should also be noted that the present design could not determine a match for *no diagnosis present* as the case vignettes always included a diagnosis. In a future naturalistic study with patients, this limitation would be removed.

Health apps are considered to be a support system rather than a substitute for doctors and psychotherapists, both by development companies and by doctors [53] and psychotherapists [5,54]. For example, a recent study [54] interviewed 720 general practitioners about future digitization in the health care system. Of them, 68% considered it unlikely that doctors would ever be replaced for diagnostic tasks. Previous findings on the appropriateness of the recommendation for further treatment vary between 33% [41] and 81% [55] agreement regarding the triage performance of the app and doctors or nurses, depending on, for example, the app used, the urgency of the treatment, and the judging person (doctor or nurse).

Combined with future research to test diagnostic accuracy, it would also be interesting to compare the extent to which differences exist when patients do the input themselves. As already mentioned, there is a clear need to catch up in the field of diagnostics in childhood and adolescence using the app tested here. Parents are often uncertain about the significance of existing symptoms, behavioral abnormalities, or developmental deficits. Even if electronic health systems are to be understood as diagnostic indications or screenings and not something that can replace a doctor or psychotherapist, such a system can provide parents with relevant information and initial instructions for action.

As the app is used particularly at the consumer level, and our pilot study indicated that diagnostic quality was lower among users from the general population and students, an important research perspective is to examine in which areas the weaknesses and deficits lie with nonprofessionals and how these can be addressed in further development. Such development could also be valuable, for example, for use in regions or countries with limited medical and psychotherapeutic care. The professional level would also benefit from a higher reliability of AI-supported diagnosis of mental disorders in childhood and adolescence. The fact that a patient is referred to an appropriate medical or psychotherapeutic specialty, for example, has relevant effects on the patient and the physician and can have considerable health economic effects.

As health apps collect and process highly sensitive health data, data security is of immense importance. Frequent shortcomings of current health apps are inadequate information about the nature and purpose of further processing of the data, missing or excessively complex data privacy statements, and comparatively easy access and manipulation by third parties [6,18,56]. Health apps should increasingly be certified based on defined catalogues of criteria and provided with a seal of quality, although this has rarely been done to date [57]. Overall, challenges remain to improve data security and the standardization of quality assurance, in particular, transparency for users, data protection control, and the handling of big data [14,36,57].

### Conclusions

Health-related apps are also widely used for mental health conditions and disorders (in the general population and increasingly by practitioners and the public health system), but little is known about the diagnostic quality of health apps for mental disorders. This pilot study found that the diagnostic agreement between the health app and the diagnosis of the case vignettes for mental disorders was overall low to moderate. The diagnostic quality was shown to be dependent on the user and the type of mental disorder. Only when psychotherapists used the app for mental disorders in adulthood, good diagnostic agreements were found. Therefore, the health app should be used with caution in the general population and should be considered as a first indication of possible mental health conditions. In particular, improvements in the app with regard to mental disorders in childhood and adolescence and further research are needed.

## Appendix

Multimedia Appendix 1 List of mental disorders.

## Abbreviations

ADHD: attention-deficit hyperactivity disorder
AI: artificial intelligence
e–mental health: electronic mental health
ICD: International Classification of Diseases
