# Supplementary material for: Accuracy of a Chatbot (Ada) in the Diagnosis of Mental Disorders: Comparative Case Study With Lay and Expert Users
Source: JMIR Form Res. 2019 Oct 29;3(4):e13863. doi: 10.2196/13863 (PMC6914276; doi:10.2196/13863)
Supplement: Multimedia Appendix 1 [file formative_v3i4e13863_app1.pdf]

Appendix

**Mental disorders in case books (reference)**	**Main diagnoses in Ada Health**	**Examples of differential diagnoses in Ada Health**
Cases with adults	*Matches in italics*	*Matches in italics*
1. Depressive episode (Stieglitz et al., 2007, p. 145)	*Depressive episode*, depressive disorder	Adjustment disorder, burnout, bipolar affective disorder
2. Social phobia (Stieglitz et al., 2007, p. 173)	*Social phobia*, generalized anxiety disorder (GAD)	GAD, panic disorder
3. Posttraumatic Stress Disorder (PTSD; Stieglitz et al., 2007, p. 207)	*PTSD*	Severe depression, intrusions
4. Borderline personality disorder (Stieglitz et al., 2007, p. 235)	*Borderline personality disorder*, PTSD	PTSD, severe depression
5. Alzheimer's dementia (Freyberger & Dilling, 2014, p. 19)	*Dementia* (Alzheimer's, vascular), partly no clear main diagnosis	Adjustment disorder, schizophrenia
6. Bipolar affective disorder (Freyberger & Dilling, 2014, p. 171)	*Bipolar affective disorder*, schizoaffective disorder	Schizoaffective disorder, schizophrenia, cyclothymia
7. Anorexia nervosa (Freyberger & Dilling, 2014, p. 179)	*Anorexia nervosa*	Bulimia nervosa, obsessive-compulsive disorder (OCD)
8. GAD (Freyberger & Dilling, 2014, p. 217)	*GAD*, adjustment disorder, depression	Depression, panic attacks/disorder, social phobia
9. Alcohol dependence (Freyberger & Dilling, 2014, p. 293)	Alcohol abuse, depression, PTSD, social phobia	Depression, GAD, PTSD
10. OCD (Freyberger & Dilling, 2014, p. 299)	*OCD*, Depression	Anorexia/bulimia nervosa, depression
11. Schizophrenia (Freyberger & Dilling, 2014, p. 307)	*Schizophrenia*, schizoaffective disorder	OCD, schizoaffective disorder

12. Undiff. Somatoform disorder (Stieglitz et al., 2007, p. 193)	Hypertension, GAD, degenerative disease of the cervical spine	Anemia, migraine, fibromyalgia
Cases with children and adolescents		
1. Selective mutism (Petermann, 2009, p. 227)	Social phobia, Asperger's syndrome	Autism-spectrum-disorder (ASD), depression, GAD
2. Enuresis (Petermann, 2009, p. 290)	*(mixed) incontinence*	Obstructive uropathy, spina bifida occulta, *(mixed) incontinence*
3. Conduct disorder (Poustka & Goor-Lambo, 2008, p. 101)	*Conduct disorder*, PTSD	Oppositional defiant disorder, borderline/narcissistic personality disorder
4. Adjustment Disorder (Poustka & Goor-Lambo, 2008, p. 153)	*Adjustment disorder*, functional abdominal pain	Depression, PTSD, GAS
5. Attention-deficit hyperactivity disorder (ADHD; Poustka & Goor-Lambo, 2008, p. 181)	*ADHD*, GAS	PTSD, depression, ASD
6. Separation anxiety disorder of childhood (Poustka & Goor-Lambo, 2008, p. 83)	Adjustment disorder, GAS, depression	Conduct disorder, depression, PTSD
7. Autism (Poustka & Goor-Lambo, 2008, p. 229)	*ASD*, Asperger's syndrome	ADHD, schizophrenia
8. Agoraphobia with panic disorder (Stieglitz et al., 2007, p. 159)	*agoraphobia, panic disorder,* specific phobia	specific phobia, panic attacks, anxiety disorder
